# Similar Responses of Circulating MicroRNAs to Acute High-Intensity Interval Exercise and Vigorous-Intensity Continuous Exercise

**DOI:** 10.3389/fphys.2016.00102

**Published:** 2016-03-18

**Authors:** Shu F. Cui, Cheng Wang, Xin Yin, Dong Tian, Qiu J. Lu, Chen Y. Zhang, Xi Chen, Ji Z. Ma

**Affiliations:** ^1^State Key Laboratory of Pharmaceutical Biotechnology, Collaborative Innovation Center of Chemistry for Life Sciences, Jiangsu Engineering Research Center for MicroRNA Biology and Biotechnology, NJU Advanced Institute for Life Sciences (NAILS), School of Life Sciences, Nanjing UniversityNanjing, China; ^2^Department of Clinical Laboratory, Jinling HospitalNanjing, China; ^3^The Lab of Military Conditioning and Motor Function Assessment, The PLA University of Science and TechnologyNanjing, China

**Keywords:** circulating microRNAs, high intensity interval exercise, biomarkers, aerobic capacity, vigorous-intensity continuous exercise

## Abstract

High-intensity interval exercise (HIIE) has been reported to be more beneficial for physical adaptation than low-to-moderate exercise intensity. Recently, it is becoming increasingly evident that circulating miRNAs (c-miRNAs) may distinguish between specific stress signals imposed by variations in the duration, modality, and type of exercise. The aim of this study is to investigate whether or not HIIE is superior to vigorous-intensity continuous exercise (VICE), which is contributing to develop effective fitness assessment. Twenty-six young males were enrolled, and plasma samples were collected prior to exercise and immediately after HIIE or distance-matched VICE. The miRNA level profiles in HIIE were initially determined using TaqMan Low Density Array (TLDA). And the differentially miRNAs levels were validated by stem-loop quantitative reverse-transcription PCR (RT-qPCR). Furthermore, these selective c-miRNAs were measured for VICE. Our results showed that some muscle-related miRNAs levels in the plasma, such as miR-1, miR-133a, miR-133b, and miR-206 significantly increased following HIIE or VICE compared to those at rest (*P* < 0.05), and there was only a significant reduction in miR-1 level for HIIE compared to VICE (*P* < 0.05), while no significant differences were observed for other muscle-related miRNAs between both exercises (*P* > 0.05). In addition, some tissue-related or unknown original miRNA levels, such as miR-485-5p, miR-509-5p, miR-517a, miR-518f, miR-520f, miR-522, miR-553, and miR-888, also significantly increased (*P* < 0.05) in both exercises compared to rest. However, no significant differences were found between both exercises (*P* > 0.05). Overall, endurance exercise assessed in this study both led to significant increases in selective c-miRNAs of comparable magnitude, suggesting that both types of endurance exercise have general stress processes. Accordingly, the similar responses to both acute exercises likely indicate both exercises can be used interchangeably. Further work is needed to reveal the functional significance and signaling mechanisms behind changes in c-miRNA turnover during exercise.

## Introduction

High-intensity interval exercise (HIIE) can take a variety of forms and is currently one of the most effective means of improving cardiorespiratory and metabolic function and, in turn, physical performance in athletes (Buchheit and Laursen, 2013b; Milanovic et al., 2015). HIIE involves repeatedly exercising at a high intensity for periods of 30 s to several minutes, separated by 1–5 min of recovery (Buchheit and Laursen, 2013a). In addition, continuous moderate-intensity aerobic exercise that is sustained for 30 min or more is also the preferred training modality for many athletes (Buchheit and Laursen, 2013b). Previous studies have shown that HIIE can have more profound effects on cardiovascular function and aerobic capacity than isocaloric low and moderate intensity exercise (VICE) in animal models (Kemi et al., 2005; Hafstad et al., 2011), healthy subjects (Gurd et al., 2010; Nybo et al., 2010) and patients in hypertension and coronary heart disease (Ciolac, 2012; Moholdt et al., 2014). HIIE offers the possibility of enhancing the performance of high-intensity exercise for far longer periods than is possible with continuous exercise (Gillen and Gibala, 2014).

Exercise has been shown to be a potent activator of gene expression, including genes encoding small non-coding RNAs called microRNAs (miRNAs), which can play a central role in the post-transcriptional regulation of gene expression for a broad range of biological processes (Kirby and McCarthy, 2013; McCarthy, 2014). miRNAs are responsive to acute aerobic and resistance exercise in the brain, blood, skeletal and cardiac muscle, adipose tissue and even buccal cells (Denham et al., 2014). The expression patterns of miRNAs vary considerably depending on the mode of exercise (Kirby and McCarthy, 2013). Some of these alterations in gene expression may be attributed to changes in the levels of several specific miRNAs in diverse tissues induced by exercise such as skeletal muscle-related miRNAs (Drummond, 2010; Nielsen et al., 2010).

Recently, it has been suggested that circulating miRNAs (c-miRNAs) may serve as physiological mediators of exercise-induced adaptation (Baggish et al., 2011; Aoi et al., 2013; Aoi and Sakuma, 2014; Mooren et al., 2014). Moreover, it is becoming increasingly evident that c-miRNAs may distinguish between specific stress signals imposed by variations in the duration, modality, and type of exercise (Nielsen et al., 2014; De Gonzalo-Calvo et al., 2015). At present, although HIIE is a well-known, time-efficient training method for improving cardiorespiratory and metabolic function (Buchheit and Laursen, 2013a,b), the acute molecular mechanisms underlying its effects have not been fully elucidated. The aim of this study is to investigate whether or not high intensity interval exercise is superior to vigorous-intensity continuous exercise (VICE), which is contributing to develop effective fitness assessment. We formulated the hypothesis that changes to miRNAs in the circulation were different in acute HIIE and VICE during exercise, which may provide insight into the potentially superior benefits of HIIE to other forms of endurance exercise.

## Materials and methods

### Subjects

Twenty-six healthy young men (age, 20.38 ± 0.12 years; height, 1.75 ± 0.01 m; body mass, 68.35 ± 1.04 kg; and BMI, 21.92 ± 0.24 kg·m^−2^) who were habituated to a regular exercise regimen were recruited to participate in this study. All subjects had performed regular endurance training for 2 years (Training ≥ 3 d·wk^−1^). None of the participants had any current or prior chronic disease, a history of smoking, or current use of any medications or dietary supplements. Written informed consent was obtained from all of the participants. Ethical approval for this study conformed to the standards of the Declaration of Helsinki, and the protocol was approved by the Institutional Review Board of Nanjing University.

### Exercise trial

A single trial of HIIE was performed 3 days following the incremental exercise test to eliminate the effect of transient metabolic changes that continue for a period of time post-exercise. The HIIE trial consisted of 7 bouts of 4 min of high-intensity running (~85–95% of HR_max_), interspersed with 2 min of active recovery in addition to 10 min of warm up and 5 min of cool down (Buchheit and Laursen, 2013a). The average running time was close to 1 h. After 3 days, a VICE trial consisting of continuous running was performed, where the distance covered matched the distance covered during the HIIE trial. Heart rate (HR) was measured during each exercise using a Polar heart rate monitor with a Polar TEAM 2 (Polar TEAM Heart Rate Monitor, Finland). The room temperature was between 19 and 22°C, and the relative humidity between 40 and 50%.

### Maximal oxygen consumption and maximal heart rate test

The maximal oxygen uptake (VO_2max_) was measured by using a portable metabolic system that measures on a breath-by-breath basis (K4b2, Cosmed, Rome, Italy), followed by an incremental exercise test on a motor-driven treadmill (h/p/cosmos quasar, Germany). Calibration procedures were performed before the test, according to the manufacturer's recommendations. The treadmill running test was performed using the Bruce protocol (Bruce, 1972). All subjects were encouraged to exercise to exhaustion. The highest 1-min average of O_2_ uptake was determined as the VO_2max_. Exhaustion was ensured by a respiratory exchange ratio >1.05. The maximal heart rate (HR_max_) was defined as the highest 5-s average during the VO_2max_ test by a Polar pulse meter.

### Plasma sampling

Blood samples were collected from the subjects at three time points: prior to acute exercise testing (Rest), immediately after the HIIE trial and after the distance-matched VICE trial (within 1 min of completion of the exercise testing). Five milliliters of venous blood was collected in standard anticoagulant (EDTA)-treated Vacutainer tubes. Immediately after each blood drawn all blood samples were centrifuged at 1500 g for 10 min to pellet the cellular elements and then centrifuged at 10,000 g for 5 min at 4°C to completely remove the cell debris. To minimize freeze-thaw degradation, the supernatant plasma was then immediately frozen at −80°C for future analysis.

### TaqMan low density array

For the TaqMan Low Density Array (TLDA), equal volumes of plasma from the 26 healthy young male subjects were pooled separately to form Rest and HIIE sample pools. RNA was extracted from each pooled sample according to a previously described protocol (Luo et al., 2013). miRNA profiling of 754 different human miRNAs was then performed using a 7900 HT Fast Real-Time PCR System (Applied Biosystems) as specified by the protocols of the manufacturer. The results were expressed as threshold cycle (Ct) values and normalized to an internal control miRNA U6 recommended by the manufacturer. The fold changes of c-miRNA were calculated using the equation 2^−ΔΔCt^.

### RNA extraction and RT-qPCR

Total RNA, including miRNA, was isolated from 100 μl of plasma, using a 1-step phenol/chloroform purification protocol (Luo et al., 2013). An exogenous reference gene, plant miRNA MIR2911 was spiked into each sample to control for variability in the RNA extraction and purification procedures (Yan et al., 2015). Specially, to standardize the volumes (100 μl) of each plasma supernatant, we added 20 fmol (20 μl total volume) of a chemically synthesized miRNA duplex mimic of MIR-2911. RT-qPCR was performed using a TaqMan PCR kit and an Applied Biosystems 7300 Sequence Detection System to quantify the abundance of mature miRNAs. After the reactions, the cycle threshold (Ct) data were evaluated using the default threshold settings, and the mean Ct was determined from the triplicate PCRs. The relative levels of miRNAs were normalized to MIR-2911 and calculated using the 2^−ΔΔCt^ method. ▵Ct was calculated by subtracting the Ct values of MIR-2911 from the average Ct values of the target miRNAs. ▵▵Ct was then determined by subtracting the ▵Ct of the rest condition from the ▵Ct of the exercise conditions.

### Statistical analyses

GraphPad Prism 5 and SigmaPlot 10.0 packages were used. All data were presented as the mean ± standard error of the mean (SEM). The distribution of the data was tested for normality using the Shapiro-Wilk normality test. The results were analyzed using the Friedman test to determine significance. When appropriate (*P* < 0.05), a Dunn's Multiple Comparison *post-hoc* test was applied to compare differences between the three groups. Correlation analyses were performed using the Spearman's or Pearson's method as appropriate for data distribution. Values of *P* < 0.05 are considered significant.

## Results

### Changes in heart rates in response to acute HIIE and VICE

**Figure 1:**
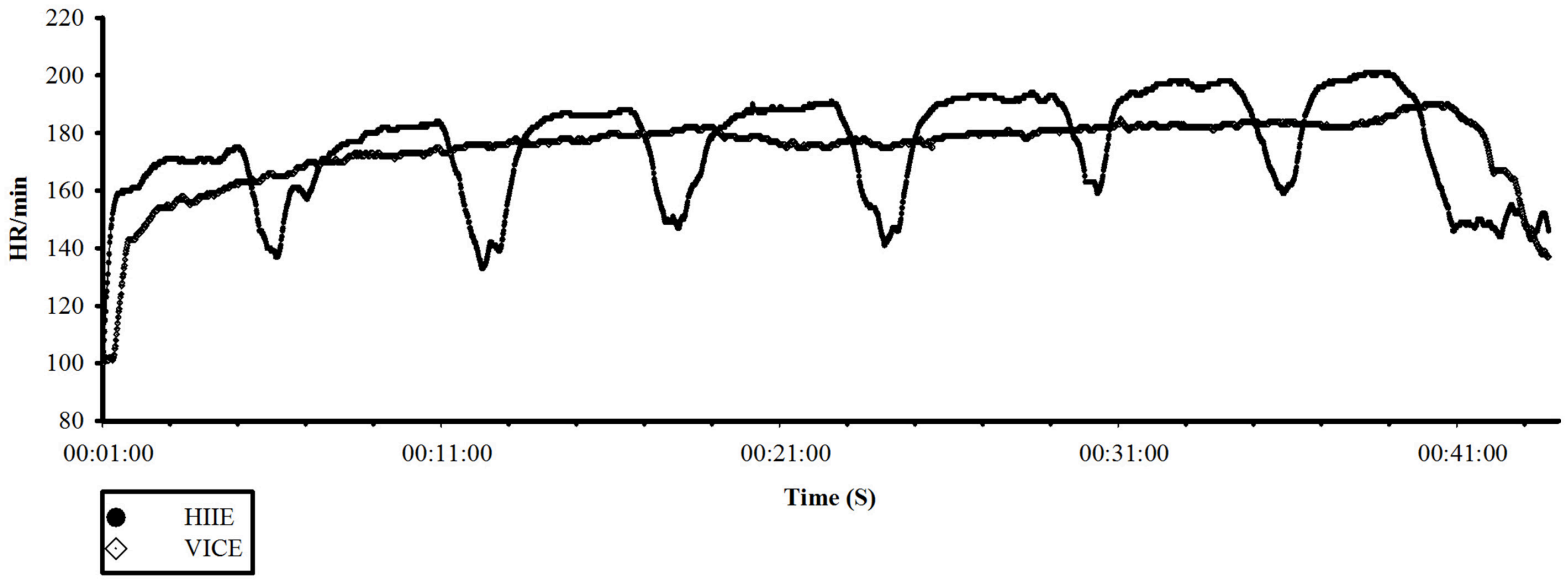
**Heart rate curves for a representative subject performing high-intensity interval exercise or vigorous-intensity continuous exercise (VICE)**.

According to the HIIE and VICE models (Figure 1), the mean running intensity of HIIE was 93% of the HR_max_, and the mean intensity of active recovery was approximately 80% of the HR_max_. The mean running intensity of VICE was 87% of the HR_max_. The running intensities of HIIE and VICE both reached the prescribed intensity zone of the 85–95% of HR_max_ (Nybo et al., 2010; Moholdt et al., 2014), and there was no significant difference in the mean HR during the entire exercise session between HIIE and VICE. However, there was a significant difference in the HR during running between the HIIE and VICE trials (*P* < 0.001). The data for the mean heart rates during the HIIE and VICE sessions are summarized in Table 1.

**Table 1:** **The mean heart rates during rest, HIIE and VICE session**.

		**HIIE**			**VICE**
**HR_*rest*_ (bpm)**	**HR_*max*_ (bpm)**	**HR_*running*_ (bpm)**	**HR_*recovery*_ (bpm)**	**HR_*whole*_ (bpm)**	**HR_*running*_ (bpm)**
72.00 ± 1.39	199.60 ± 0.17	185.83 ± 1.46^***^	160.88 ± 1.21	176.47 ± 1.86	175.29 ± 1.31

*Values represent as the mean ± SEM (n = 10)*.

*** *Indicates differences compared to VICE, P < 0.001*.

### Global screening of circulating miRNAs in response to HIIE

A TLDA analysis to screen and select candidate miRNAs were first employed. miRNAs were considered to be differently changed if their Ct values were <40 and if there was a at least two-fold difference in the HIIE sample compared to the Rest controls. miRNA profiling analysis revealed that 229 plasma miRNAs were increased and 220 plasma miRNAs were decreased following HIIE. Fifty-seven of the markedly altered miRNAs from the HIIE group (fold-change > 20) were listed in Supplementary Table 1. Furthermore, some muscle-specific miRNAs, such as miR-1, miR-133a, miR-133b, and miR-206, were selected (Boettger et al., 2014). Other tissue-related or unknown originated miRNAs were then selected as candidate miRNAs for further testing by RT-qPCR, including miR-485-5p, miR-509-5p, miR-517a, miR-518f, miR-520f, miR-522, miR-553, and miR-888.

### Confirmation of the candidate circulating miRNAs by RT-qPCR

**Figure 2:**
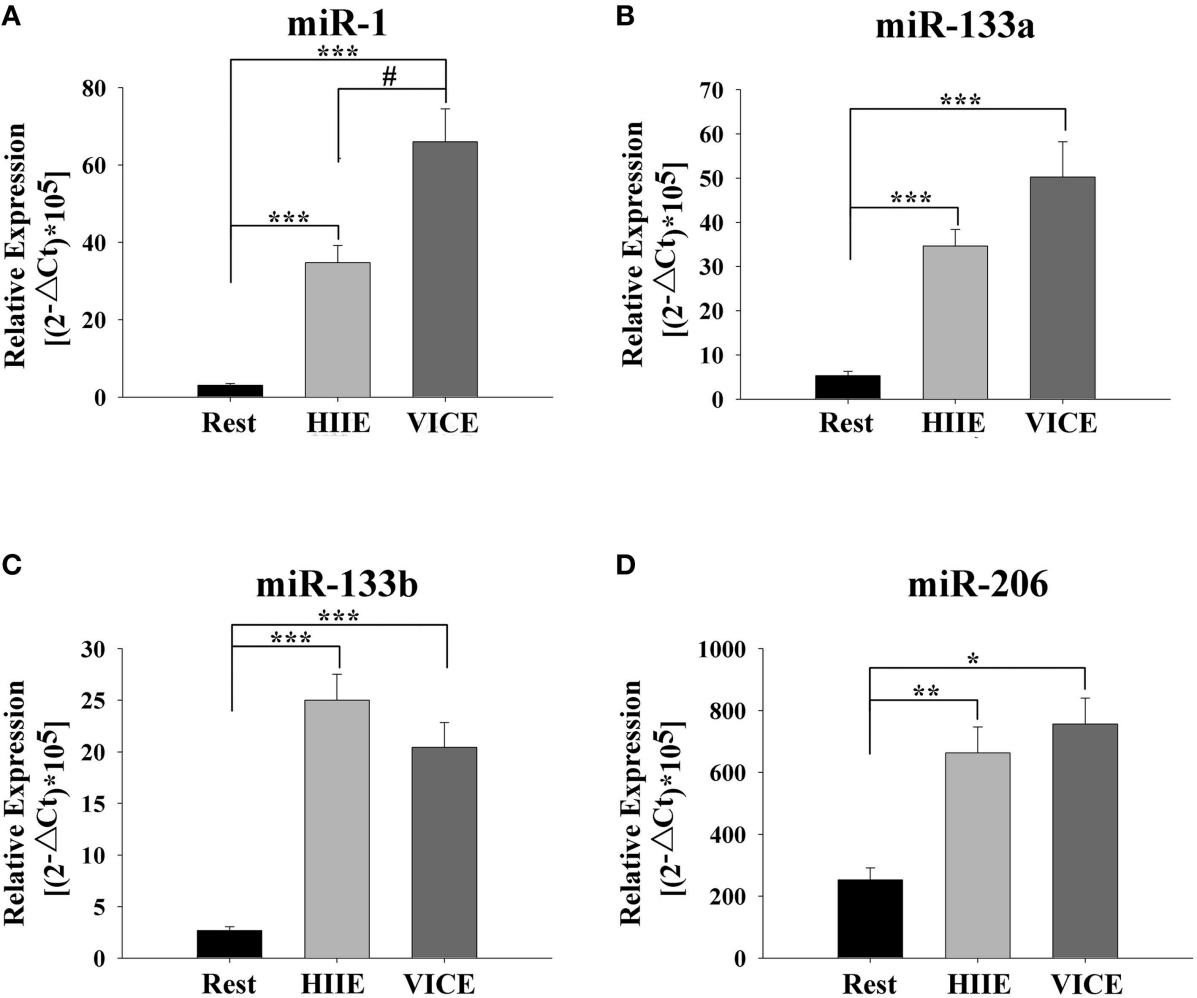
**Muscle-specific c-miRNAs are elevated after high intensity interval exercise and vigorous intensity continuous exercise**. Immediately after high-intensity interval exercise (HIIE) and vigorous-intensity continuous exercise (VICE), plasma levels of miR-1 **(A)**, miR-133a **(B)**, miR-133b **(C)**, miR-206 **(D)** were significantly increased. Compared to the HIIE, plasma level of miR-1 in the VICE was increased significantly. c-miRNA levels are displayed as relative levels based on the formula (2^−▵Ct^×10^5^). Values represent the mean ± SEM obtained from 26 subjects. ^*^Indicates differences compared to Rest, *P* < 0.05; ^**^Indicates differences compared to Rest, *P* < 0.01; ^***^Indicates differences compared to Rest, *P* < 0.001; ^#^Indicates differences compared to VICE, *P* < 0.05.

**Figure 3:**
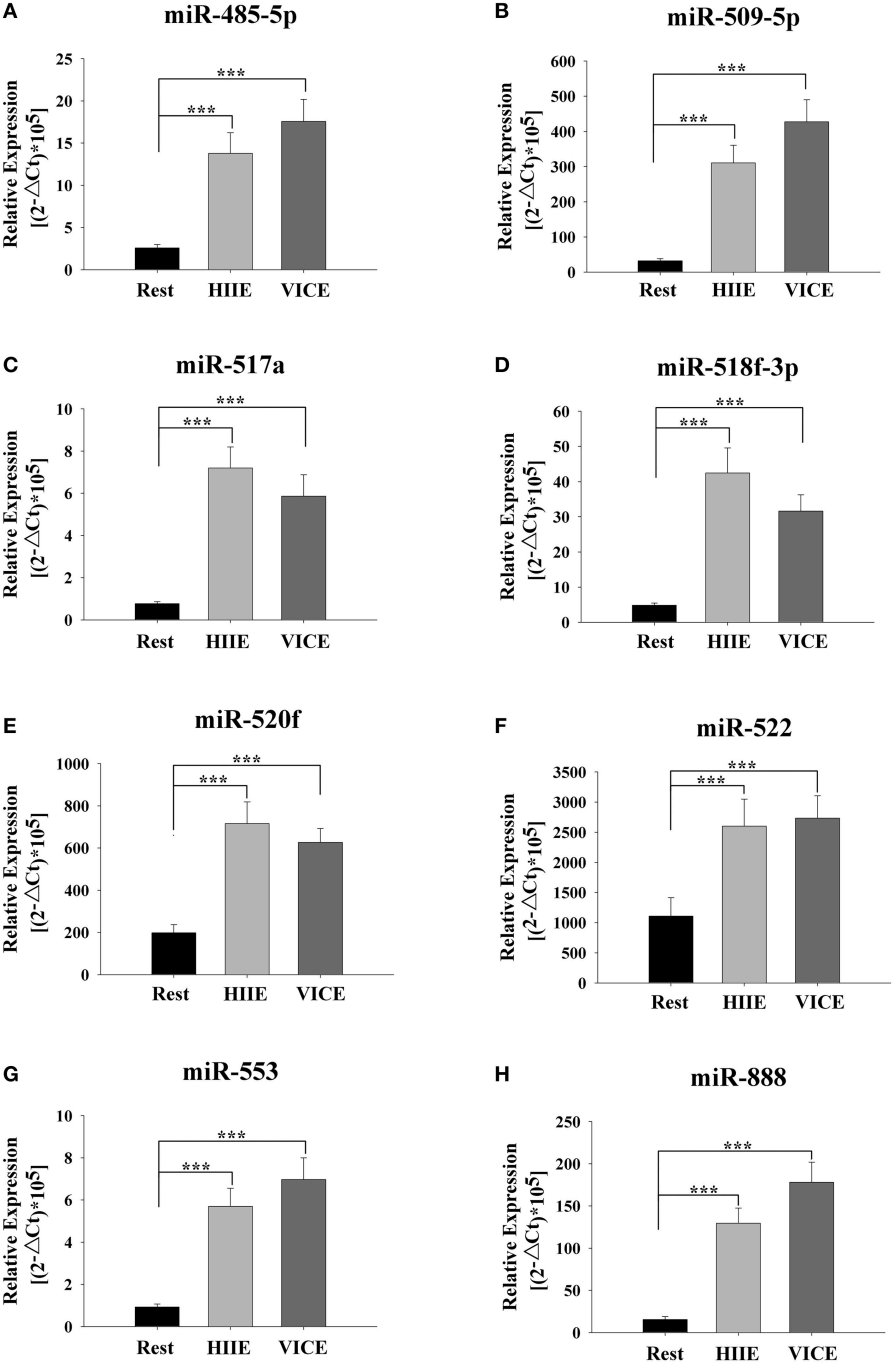
**Non-muscle original c-miRNAs are elevated following high intensity interval exercise and vigorous intensity continuous exercise**. Some tissue-related **(A–F,H)** or unknown originated **(G)** miRNAs in plasma were significantly increased following high intensity interval exercise (HIIE) and vigorous intensity continuous exercise (VICE). c-miRNA levels are displayed as relative levels based on the formula (2^−▵Ct^ × 10^5^). Values represent the mean ± SEM obtained from 26 subjects. ^***^Indicates differences compared to Rest, *P* < 0.001.

A TaqMan probe-based RT-qPCR assay to confirm the change of the candidate miRNAs in individual plasma samples was next employed. The muscle-specific miRNAs levels in the plasma, such as miR-1, miR-133a, miR-133b, and miR-206 significantly increased in HIIE compared to rest (*P* < 0.05) (Figure 2). In addition, some selective tissue-related or unknown originated miRNAs levels in plasma, such as miR-485-5p, miR-509-5p, miR-517a, miR-518f, miR-520f, miR-522, miR-553, and miR-888, were also significantly increased in HIIE compared to rest (*P* < 0.05) (Figure 3).

### Changes in circulating miRNAs between acute HIIE and VICE

To assess the difference between the selected plasma miRNAs in discriminating between the two types of training, these c-miRNAs levels between HIIE and VICE were compared. No significant differences were found in miR-133a, miR-133b, and miR-206 level in the plasma between HIIE and VICE (*P*>0.05) (Figure 2). However, the miR-1 level in VICE increased significantly compare to HIIE between HIIE and VICE (*P* < 0.05) (Figure 2). There were no significant differences in the levels of plasma miR-485-5p, miR-509-5p, miR-517a, miR-518f, miR-520f, miR-522, miR-553, and miR-888 between HIIE and VICE (*P*>0.05) (Figure 3).

### Association between circulating miRNA levels and peak oxygen consumption

To explore the feasibility of using c-miRNAs as potential biomarkers for aerobic capacity, the correlation between the changes in the plasma levels of these selected miRNAs and VO_2max_ were analyzed. No correlations were found between the changes in these selected c-miRNAs and the VO_2max_ (*r*-values between −0.391 and 0.252, *P*>0.05).

## Discussion

It is well documented that each tissue exhibits a particular miRNA expression profile, suggesting specific functions (Schneeberger et al., 2015). Our results suggested that the similar physiological demands of both types of endurance exercise influenced the c-miRNAs responses to these exercise bouts. The change of c-miRNAs in response to endurance exercises likely suggests their important role in evaluating specific adaptations induced by exercises, which might, in turn, have an impact on adaptation to training.

Aerobic exercise is typically characterized by lower intensity, longer-duration contractile activity which has long been tied to good health (Rowe et al., 2014). A growing body of evidence demonstrates that HIIE can serve as an effective alternate to traditional endurance-based training (Buchheit and Laursen, 2013b; Milanovic et al., 2015). The mechanisms involved in this phenomenon are not yet fully understood. During endurance exercise, numerous molecular cascades in distant tissues are affected to handle the stress. The study of c-miRNAs may provide additional insight into the internal stress experienced by the individual and the effects of exercise.

In the present study, the level of some cardiac and skeletal muscle-enriched miRNAs in the plasma, which were increased in several prior studies following a full or half-marathon run (Gomes et al., 2014; Mooren et al., 2014; Uhlemann et al., 2014), such as miR-1, miR-133a, miR-133b, and miR-206 were also significantly increased during HIIE and VICE. Both types of training can effectively improve cardiac and skeletal muscle metabolic function (Buchheit and Laursen, 2013a). However, the level of miR-1 in the plasma in VICE showed a significant increase compared to HIIE, suggesting that VICE may place more stress on the heart or skeletal muscle. For a long time, the basic core concept of specificity of training is based in motor unit recruitment (Kraemer and Szivak, 2012) with the understanding that the non-activated muscle tissue remains essentially untrained (Kraemer and Szivak, 2012). In contrast, high-intensity exercise combines greater recruitment of type II muscle fibers and a greater stimulation rate of already recruited fibers (Sale, 1987). Since during competitive games players have often more space to run and reach higher running speeds (up to 85–90% of maximal sprinting speed (Di Salvo et al., 2010; Mendez-Villanueva et al., 2011) for likely similar metabolic demands (Buchheit and Laursen, 2013a). There are likely several approaches (i.e., HIIE format) that, considered in isolation, will achieve a similar metabolic and/or neuromuscular training adaptation outcome (Buchheit and Laursen, 2013a). In our study, no changes for muscle enriched c-miRNA levels between HIIE and VICE, such as miR-133a, miR-133b, and miR-206, may reflect similar physical demands for peripheral components. At present, several hundred miRNAs are detectable in skeletal muscle (McGregor et al., 2014), which limits our ability to accurately estimate HIIE sessions. However, it is likely that acute neuromuscular load may be greater with HIIE.

Eight miRNAs not restricted to muscle origin also significantly increased in plasma during this study of HIIE and VICE, suggesting that numerous acute transient responses occur to enable the body to cope with different stressors, such as thermal, metabolic, hypoxic, oxidative, and mechanical stress. However, no significant differences in eight miRNAs levels were found between HIIE and VICE, indicating that they may have shared a common response to exercise. The benefit of exercise can be found in other tissues, which can prevent or effectively treat a multitude of degenerative conditions, including cardiovascular disease, cancer, diabetes, depression, and many others (Watson and Baar, 2014). Non-muscle tissues show exercise-induced changes including adipose, liver, lung, endothelium, bone and hence may release miRNAs into circulation (McGregor et al., 2014). For example, miR-517a is closely associated with nuclear factor kappa-B (NF-κB) signaling, is conserved, ubiquitously expressed, and a pivotal regulator of immune response, inflammation and cell survival (Olarerin-George et al., 2013); The miR-888 cluster family, exclusively expressed in the reproductive system of human and non-human primates, is associated with epididymal physiology and immune cell functions (Belleannee, 2015); miR-518f was preferentially expressed in the bones of osteoporotic patients (Garmilla-Ezquerra et al., 2015). Some miRNAs, such as miR-509-5p (Ren et al., 2014), miR-485-5p (Sun et al., 2015), miR-520f (Harvey et al., 2015), and miR-522 (Zhang et al., 2015), are normally associated with pathology of the cancers. The source and function of miR-553 is not clear.

Taken together, most of the c-miRNAs identified in plasma had a similar response to both exercises. Moreover, the nature and magnitude of the responses of the c-miRNAs were influenced by exercise type, which likely depends on exercise intensity or volume (De Gonzalo-Calvo et al., 2015). Given that the same average intensity and distance for both conditions, it is likely that the c-miRNAs response might be more closely related to the relative than absolute metabolic demands. The identical effects on c-miRNAs likely suggest both exercises might represent a similar effective and potentially promoting body health.

At present, the mechanism of exercise induced c-miRNAs elevation is likely much more complex. A number of plausible, perhaps complementary suggestions have been proposed, such as muscle cell damage (Gomes et al., 2014; Uhlemann et al., 2014), de novo miRNA transcription (Baggish et al., 2014; Gomes et al., 2014; Mooren et al., 2014) and activate miRNAs secretory (Aoi and Sakuma, 2014; Gomes et al., 2014; Mooren et al., 2014). The miRNA pool in the circulation might be determined by a balance between cellular uptake/release and intracellular catabolism and anabolism during exercise. A recent study demonstrated that cells secrete extracellular vesicles (EVs), which carry proteins characteristic of exosomes, with a size of 100–130 nm, that can be released into the circulation following an incremental exercise protocol of cycling or running until exhaustion (Fruhbeis et al., 2015), likely suggesting that c-miRNAs, as well as other circulating factors, e.g., hormones, adipokines, and myokines, may be associated with exercise-induced benefits (Aoi, 2015). Thus, changed muscle-specific miRNAs levels in plasma/serum might be useful as molecular biomarkers for monitoring physiology related body conditions. For example, although no correlations were found between the change in the c-miRNA level and VO_2max_ in our study, a correlation exists between aerobic exercise performance and the change of specific miRNAs in circulating blood (Denham et al., 2014), such as miR-1, miR-133a, and miR-206 (Mooren et al., 2014).

Furthermore, the biological significance of exercise-induced c-miRNAs also remains elusive. Recently, studies suggested a possible role in cell-to-cell communication, where c-miRNAs might be able to mediate gene expression in target tissues in a way comparable to hormones and cytokines (Russell and Lamon, 2015). During exercise, many systems are responsive to exercise, including skeletal muscle, immune, the cardiovascular and skeletal system. The corresponding miRNAs changes in plasma have been observed in our study. It is unknown whether changes in circulating miRNAs during exercise directly reflect changes in muscle miRNA expression. Some muscle-related miRNAs, such as miR-1, miR-133a, miR-133b, are also increased in human skeletal muscle during exercise (Nielsen et al., 2010; Russell et al., 2013). Moreover, data from tracer studies in rodents and humans show that global protein synthesis, involved in energy metabolism accompanied with alterations of heat shock and proteasomal proteins, is blunted in working skeletal muscle (Rose and Richter, 2009; Schild et al., 2015). Thus, we speculate that some specific c-miRNAs increase during exercise might reflect the blunting of protein synthesis be controlled by miRNAs as the upstream signaling molecular. Additional study requires testifying this hypothesis.

Altogether, our results suggest the ability of exercise to increase some specific miRNAs in the circulation might underlie the subsequently beneficial effects of exercise on tissue function. Additionally, identification of specific molecular biomarkers in the form of miRNAs might be suitable for monitoring training interventions.

## Author contributions

SC designed the study, performed the experiments, collected and analyzed the data, and revised the final version of the manuscript. XY, DT, and QL recruited participants, collected samples and performed the experiments. CW, CZ, and XC designed the study and critically revised the final version of the manuscript. JM designed the study, recruited participants, analyzed the data and wrote the final version of the manuscript. All authors read and approved the final version of the manuscript.

## Funding

The authors acknowledge that this work was supported by the National Basic Research Program of China (2014CB542300), the National Natural Science Foundation of China (81101330, 31271378, 81250044), the Natural Science Foundation of Jiangsu Province (BK2012014) and the Research Special Fund for Public Welfare Industry of Health (201302018). This work was also supported by the Program for New Century Excellent Talents in University from Ministry of Education of China (NCET-12-0261).

### Conflict of interest statement

The authors declare that the research was conducted in the absence of any commercial or financial relationships that could be construed as a potential conflict of interest.

## References

[B1] Aoi W. (2015). Frontier impact of microRNAs in skeletal muscle research: a future perspective. Front. Physiol. 5:495 10.3389/fphys.2014.00495 PMC4283715 25601837

[B2] Aoi W. Ichikawa H. Mune K. Tanimura Y. Mizushima K. Naito Y. (2013). Muscle-enriched microRNA miR-486 decreases in circulation in response to exercise in young men. Front. Physiol. 4:80 10.3389/fphys.2013.00080 PMC3622901 23596423

[B3] Aoi W. Sakuma K. (2014). Does regulation of skeletal muscle function involve circulating microRNAs? Front. Physiol. 5:39 10.3389/fphys.2014.00039 PMC3925823 24596559

[B4] Baggish A. L. Hale A. Weiner R. B. Lewis G. D. Systrom D. Wang F.. (2011). Dynamic regulation of circulating microRNA during acute exhaustive exercise and sustained aerobic exercise training. J. Physiol. 589, 3983–3994. 10.1113/jphysiol.2011.213363 21690193 PMC3179997

[B5] Baggish A. L. Park J. Min P. K. Isaacs S. Parker B. A. Thompson P. D.. (2014). Rapid upregulation and clearance of distinct circulating microRNAs after prolonged aerobic exercise. J. Appl. Physiol. (1985) 116, 522–531. 10.1152/japplphysiol.01141.2013 24436293 PMC3949215

[B6] Belleannee C. (2015). Extracellular microRNAs from the epididymis as potential mediators of cell-to-cell communication. Asian J. Androl. 17, 730–736. 10.4103/1008-682x.155532 26178395 PMC4577581

[B7] Boettger T. Wust S. Nolte H. Braun T. (2014). The miR-206/133b cluster is dispensable for development, survival and regeneration of skeletal muscle. Skelet. Muscle 4, 23. 10.1186/s13395-014-0023-5 25530839 PMC4272821

[B8] Bruce R. A. (1972). Multi-stage treadmill test of maximal and sub maximal exercise, in Exercise Testing and Training of Apparently Healthy Individuals: A Handbook for Physicians, ed American Heart Association (New York, NY: American Heart Association), 32–34.

[B9] Buchheit M. Laursen P. B. (2013a). High-intensity interval training, solutions to the programming puzzle. Part II: anaerobic energy, neuromuscular load and practical applications. Sports Med. 43, 927–954. 10.1007/s40279-013-0066-5 23832851

[B10] Buchheit M. Laursen P. B. (2013b). High-intensity interval training, solutions to the programming puzzle: Part I: cardiopulmonary emphasis. Sports Med. 43, 313–338. 10.1007/s40279-013-0029-x 23539308

[B11] Ciolac E. G. (2012). High-intensity interval training and hypertension: maximizing the benefits of exercise? Am. J. Cardiovasc. Dis. 2, 102–110. 22720199 PMC3371620

[B12] De Gonzalo-Calvo D. Davalos A. Montero A. Garcia-Gonzalez A. Tyshkovska I. Gonzalez-Medina A.. (2015). Circulating inflammatory miRNA signature in response to different doses of aerobic exercise. J. Appl. Physiol. (1985) 119, 124–134. 10.1152/japplphysiol.00077.2015 25997943

[B13] Denham J. Marques F. Z. O'Brien B. J. Charchar F. J. (2014). Exercise: putting action into our epigenome. Sports Med. 44, 189–209. 10.1007/s40279-013-0114-1 24163284

[B14] Di Salvo V. Baron R. Gonzalez-Haro C. Gormasz C. Pigozzi F. Bachl N. (2010). Sprinting analysis of elite soccer players during European Champions League and UEFA Cup matches. J. Sports Sci. 28, 1489–1494. 10.1080/02640414.2010.521166 21049314

[B15] Drummond M. J. (2010). MicroRNAs and exercise-induced skeletal muscle adaptations. J. Physiol. 588, 3849–3850. 10.1113/jphysiol.2010.198218 20952375 PMC3000575

[B16] Fruhbeis C. Helmig S. Tug S. Simon P. Kramer-Albers E. M. (2015). Physical exercise induces rapid release of small extracellular vesicles into the circulation. J. Extracell. Vesicles 4:28239. 10.3402/jev.v4.28239 26142461 PMC4491306

[B17] Garmilla-Ezquerra P. Sanudo C. Delgado-Calle J. Perez-Nunez M. I. Sumillera M. Riancho J. A. (2015). Analysis of the bone microRNome in osteoporotic fractures. Calcif. Tissue Int. 96, 30–37. 10.1007/s00223-014-9935-7 25432767

[B18] Gillen J. B. Gibala M. J. (2014). Is high-intensity interval training a time-efficient exercise strategy to improve health and fitness? Appl. Physiol. Nutr. Metab. 39, 409–412. 10.1139/apnm-2013-0187 24552392

[B19] Gomes C. P. Oliveira G. P. Jr. Madrid B. Almeida J. A. Franco O. L. Pereira R. W. (2014). Circulating miR-1, miR-133a, and miR-206 levels are increased after a half-marathon run. Biomarkers 19, 585–589. 10.3109/1354750X.2014.952663 25146754

[B20] Gurd B. J. Perry C. G. Heigenhauser G. J. Spriet L. L. Bonen A. (2010). High-intensity interval training increases SIRT1 activity in human skeletal muscle. Appl. Physiol. Nutr. Metab. 35, 350–357. 10.1139/H10-030 20555380

[B21] Hafstad A. D. Boardman N. T. Lund J. Hagve M. Khalid A. M. Wisloff U.. (2011). High intensity interval training alters substrate utilization and reduces oxygen consumption in the heart. J. Appl. Physiol. (1985) 111, 1235–1241. 10.1152/japplphysiol.00594.2011 21836050

[B22] Harvey H. Piskareva O. Creevey L. Alcock L. C. Buckley P. G. O'Sullivan M. J.. (2015). Modulation of chemotherapeutic drug resistance in neuroblastoma SK-N-AS cells by the neural apoptosis inhibitory protein and miR-520f. Int. J. Cancer 136, 1579–1588. 10.1002/ijc.29144 25137037

[B23] Kemi O. J. Haram P. M. Loennechen J. P. Osnes J. B. Skomedal T. Wisloff U.. (2005). Moderate vs. high exercise intensity: differential effects on aerobic fitness, cardiomyocyte contractility, and endothelial function. Cardiovasc. Res. 67, 161–172. 10.1016/j.cardiores.2005.03.010 15949480

[B24] Kirby T. J. McCarthy J. J. (2013). MicroRNAs in skeletal muscle biology and exercise adaptation. Free Radic. Biol. Med. 64, 95–105. 10.1016/j.freeradbiomed.2013.07.004 23872025 PMC4867469

[B25] Kraemer W. J. Szivak T. K. (2012). Strength training for the warfighter. J. Strength Cond. Res. 26(suppl. 2), S107–S118. 10.1519/jsc.0b013e31825d8263 22643142

[B26] Luo Y. Wang C. Chen X. Zhong T. Cai X. Chen S.. (2013). Increased serum and urinary microRNAs in children with idiopathic nephrotic syndrome. Clin. Chem. 59, 658–666. 10.1373/clinchem.2012.195297 23344497

[B27] McCarthy J. J. (2014). microRNA and skeletal muscle function: novel potential roles in exercise, diseases, and aging. Front. Physiol. 5:290 10.3389/fphys.2014.00290 PMC4116776 25132822

[B28] McGregor R. A. Poppitt S. D. Cameron-Smith D. (2014). Role of microRNAs in the age-related changes in skeletal muscle and diet or exercise interventions to promote healthy aging in humans. Ageing Res. Rev. 17, 25–33. 10.1016/j.arr.2014.05.001 24833328

[B29] Mendez-Villanueva A. Buchheit M. Simpson B. Peltola E. Bourdon P. (2011). Does on-field sprinting performance in young soccer players depend on how fast they can run or how fast they do run? J. Strength Cond. Res. 25, 2634–2638. 10.1519/JSC.0b013e318201c281 21768891

[B30] Milanovic Z. Sporis G. Weston M. (2015). Effectiveness of high-intensity interval training (HIT) and continuous endurance training for VO2max improvements: a systematic review and meta-analysis of controlled trials. Sports Med. 45, 1469–1481. 10.1007/s40279-015-0365-0 26243014

[B31] Moholdt T. Madssen E. Rognmo O. Aamot I. L. (2014). The higher the better? Interval training intensity in coronary heart disease. J. Sci. Med. Sport 17, 506–510. 10.1016/j.jsams.2013.07.007 23938444

[B32] Mooren F. C. Viereck J. Kruger K. Thum T. (2014). Circulating microRNAs as potential biomarkers of aerobic exercise capacity. Am. J. Physiol. Heart Circ. Physiol. 306, H557–H563. 10.1152/ajpheart.00711.2013 24363306 PMC3920240

[B33] Nielsen S. Akerstrom T. Rinnov A. Yfanti C. Scheele C. Pedersen B. K.. (2014). The miRNA plasma signature in response to acute aerobic exercise and endurance training. PLoS ONE 9:e87308. 10.1371/journal.pone.0087308 24586268 PMC3929352

[B34] Nielsen S. Scheele C. Yfanti C. Akerstrom T. Nielsen A. R. Pedersen B. K.. (2010). Muscle specific microRNAs are regulated by endurance exercise in human skeletal muscle. J. Physiol. 588, 4029–4037. 10.1113/jphysiol.2010.189860 20724368 PMC3000590

[B35] Nybo L. Sundstrup E. Jakobsen M. D. Mohr M. Hornstrup T. Simonsen L.. (2010). High-intensity training versus traditional exercise interventions for promoting health. Med. Sci. Sports Exerc. 42, 1951–1958. 10.1249/MSS.0b013e3181d99203 20195181

[B36] Olarerin-George A. O. Anton L. Hwang Y. C. Elovitz M. A. Hogenesch J. B. (2013). A functional genomics screen for microRNA regulators of NF-kappaB signaling. BMC Biol. 11:19. 10.1186/1741-7007-11-19 23448136 PMC3621838

[B37] Ren Z. J. Nong X. Y. Lv Y. R. Sun H. H. An P. P. Wang F.. (2014). Mir-509-5p joins the Mdm2/p53 feedback loop and regulates cancer cell growth. Cell Death Dis. 5:e1387. 10.1038/cddis.2014.327 25144722 PMC4454302

[B38] Rose A. J. Richter E. A. (2009). Regulatory mechanisms of skeletal muscle protein turnover during exercise. J. Appl. Physiol. (1985) 106, 1702–1711. 10.1152/japplphysiol.91375.2008 19074568

[B39] Rowe G. C. Safdar A. Arany Z. (2014). Running forward: new frontiers in endurance exercise biology. Circulation 129, 798–810. 10.1161/CIRCULATIONAHA.113.001590 24550551 PMC3981549

[B40] Russell A. P. Lamon S. (2015). Exercise, Skeletal Muscle and Circulating microRNAs. Prog. Mol. Biol. Transl. Sci. 135, 471–496. 10.1016/bs.pmbts.2015.07.018 26477927

[B41] Russell A. P. Lamon S. Boon H. Wada S. Guller I. Brown E. L.. (2013). Regulation of miRNAs in human skeletal muscle following acute endurance exercise and short-term endurance training. J. Physiol. 591, 4637–4653. 10.1113/jphysiol.2013.255695 23798494 PMC3784204

[B42] Sale D. G. (1987). Influence of exercise and training on motor unit activation. Exerc. Sport Sci. Rev. 15, 95–151. 10.1249/00003677-198700150-00008 3297731

[B43] Schild M. Ruhs A. Beiter T. Zugel M. Hudemann J. Reimer A.. (2015). Basal and exercise induced label-free quantitative protein profiling of m. vastus lateralis in trained and untrained individuals. J. Proteomics 122, 119–132. 10.1016/j.jprot.2015.03.028 25857276

[B44] Schneeberger M. Gomez-Valades A. G. Ramirez S. Gomis R. Claret M. (2015). Hypothalamic miRNAs: emerging roles in energy balance control. Front. Neurosci. 9:41. 10.3389/fnins.2015.00041 25729348 PMC4325937

[B45] Sun X. Liu Y. Li M. Wang M. Wang Y. (2015). Involvement of miR-485-5p in hepatocellular carcinoma progression targeting EMMPRIN. Biomed. Pharmacother. 72, 58–65. 10.1016/j.biopha.2015.04.008 26054676

[B46] Uhlemann M. Mobius-Winkler S. Fikenzer S. Adam J. Redlich M. Mohlenkamp S.. (2014). Circulating microRNA-126 increases after different forms of endurance exercise in healthy adults. Eur. J. Prev. Cardiol. 21, 484–491. 10.1177/2047487312467902 23150891

[B47] Watson K. Baar K. (2014). mTOR and the health benefits of exercise. Semin. Cell Dev. Biol. 36, 130–139. 10.1016/j.semcdb.2014.08.013 25218794

[B48] Yan Y. Shi Y. Wang C. Guo P. Wang J. Zhang C. Y.. (2015). Influence of a high-altitude hypoxic environment on human plasma microRNA profiles. Sci. Rep. 5:15156. 10.1038/srep15156 26468998 PMC4606833

[B49] Zhang S. Zhang H. Zhu J. Zhang X. Liu Y. (2015). MiR-522 contributes to cell proliferation of human glioblastoma cells by suppressing PHLPP1 expression. Biomed. Pharmacother. 70, 164–169. 10.1016/j.biopha.2015.01.017 25776496

