# Supplementary material for: Similar Responses of Circulating MicroRNAs to Acute High-Intensity Interval Exercise and Vigorous-Intensity Continuous Exercise
Source: Front Physiol. 2016 Mar 18;7:102. doi: 10.3389/fphys.2016.00102 (PMC4796030; doi:10.3389/fphys.2016.00102)
Supplement: Supplementary file 1 [file Table1.DOCX]

***Supplementary Material***

**High-intensity interval training’s beneficial effects in healthy young men: associations with specific circulating microRNAs during exercise**

**Shu Fang Cui^1^, Cheng Wang^1,2^, Xin Yin^3^, Dong Tian^3^, Qiu Ju Lu^3^, Xia Guo^3^, Chen Yu Zhang^1*^, Xi Chen^1*^, Ji Zheng Ma^1,3*^**

***Correspondence**:

Ji Zheng Ma

Email: mjz_mjj @163.com

**Supplementary Table 1**

Markedly altered miRNAs in pooled plasma samples from the HIIT group compared with those from the Rest group as determined by TLDA.

|  | △Ct | |  |
| --- | --- | --- | --- |
| miRNA | Rest | HIIE | Fold Change |
| **Increased** |  |  |  |
| miR-522-3p | 24.49 | -3.78 | 322012488.5 |
| miR-888-5p | 24.49 | 9.21 | 39581.18 |
| miR-509-5p | 24.49 | 9.5 | 32479.71 |
| miR-553 | 23.68 | 9.4 | 19871.08 |
| miR-520f | 24.49 | 11.67 | 7182.45 |
| miR-485-5p | 24.49 | 12.21 | 4969.57 |
| miR-21-3p | 23.68 | 12.67 | 2054.15 |
| miR-216b | 24.49 | 13.72 | 1738.12 |
| miR-943 | 23.68 | 13.16 | 1468.57 |
| miR-770-5p | 23.68 | 13.26 | 1362.74 |
| miR-542-5p | 24.49 | 15.83 | 404.01 |
| miR-643 | 23.68 | 15.18 | 360.22 |
| miR-302d-3p | 23.68 | 15.25 | 343.79 |
| miR-330-5p | 24.49 | 16.39 | 272.79 |
| miR-520a-3p | 24.49 | 16.75 | 213.66 |
| let-7a-3p | 23.68 | 16.01 | 203 |
| miR-630 | 23.68 | 16.07 | 195.43 |
| miR-499a-5p | 24.49 | 17.02 | 177.26 |
| miR-508-3p | 24.49 | 17.02 | 177.01 |
| miR-672 | 24.49 | 17.11 | 166.31 |
| miR-518e-3p | 22.63 | 15.44 | 146.09 |
| miR-520g | 24.49 | 17.7 | 110.16 |
| miR-653 | 24.49 | 17.75 | 106.34 |
| miR-551a | 23.68 | 17.08 | 96.52 |
| miR-374b-3p | 23.68 | 17.41 | 77.01 |
| miR-635 | 23.68 | 17.49 | 72.67 |
| miR-623 | 23.68 | 17.65 | 65.04 |
| miR-34b-5p | 23.68 | 17.77 | 60.11 |
| miR-200a-5p | 23.68 | 17.78 | 59.64 |
| miR-432-3p | 23.68 | 17.83 | 57.41 |
| miR-581 | 23.68 | 18.04 | 49.83 |
| miR-641 | 21.72 | 16.1 | 49.28 |
| miR-515-5p | 24.49 | 19.05 | 43.27 |
| miR-125b-1-3 | 23.68 | 18.27 | 42.29 |
| miR-1289 | 23.68 | 18.32 | 40.89 |
| miR-518f-3p | 3.97 | -1.01 | 31.53 |
| miR-1294 | 23.68 | 18.85 | 28.38 |
| miR-548e | 21.87 | 17.16 | 26.13 |
| miR-570-3p | 24.49 | 20 | 22.4 |
| miR-517a-3p | 13.86 | 9.41 | 21.87 |
| **Decreased** |  |  |  |
| miR-519b-3p | 2.58 | 6.91 | 0.04968 |
| miR-603 | 18.67 | 23.18 | 0.04396 |
| miR-548a-3p | 19.47 | 25.25 | 0.01823 |
| miR-520b | 19 | 25.25 | 0.01317 |
| miR-622 | 18.35 | 24.85 | 0.01109 |
| miR-32-5p | 18.7 | 25.25 | 0.01074 |
| miR-1272 | 18.12 | 24.85 | 0.00942 |
| miR-1265 | 17.52 | 24.85 | 0.00622 |
| miR-155-3p | 17.37 | 24.85 | 0.0056 |
| miR-208b | 17.31 | 25.25 | 0.00409 |
| miR-218-2-3p | 16.73 | 24.85 | 0.0036 |
| miR-639 | 16.72 | 24.85 | 0.00357 |
| miR-556-3p | 15.88 | 25.25 | 0.00152 |
| miR-580 | 15.18 | 24.85 | 0.00122 |
| miR-922 | 15.11 | 24.85 | 0.00117 |
| miR-1248 | 12.95 | 22.74 | 0.00114 |
| miR-147 | 13.2 | 25.25 | 0.00024 |
| miR-499-3p | 7.76 | 25.25 | 5.46E-06 |
